# Measurements of Inferior Vena Cava Diameter for Prediction of Hypotension and Bradycardia during Spinal Anesthesia in Spontaneously Breathing Patients during Elective Knee Joint Replacement Surgery

**DOI:** 10.3390/medicina54030049

**Published:** 2018-07-12

**Authors:** Asta Mačiulienė, Arūnas Gelmanas, Inna Jaremko, Ramūnas Tamošiūnas, Alfredas Smailys, Andrius Macas

**Affiliations:** 1 Department of Anaesthesiology, Medical Academy, Lithuanian University of Health Sciences, Eivenių 2, LT-50009 Kaunas, Lithuania; arunas.gelmanas@lsmuni.lt (A.G.); inna.jaremko@fc.lsmuni.lt (I.J.); ramunas.tamosiunas@lsmuni.lt (R.T.); andrius.macas@lsmuni.lt (A.M.); 2 Department of Orthopaedics and Traumatology, Medical Academy, Lithuanian University of Health Sciences, Eivenių 2, LT-50009 Kaunas, Lithuania; alfredas.smailys@lsmuni.lt

**Keywords:** spinal anesthesia, hypotension, bradycardia, intravascular volume, inferior vena cava

## Abstract

*Background and objective*: Hypotension and bradycardia are the most common hemodynamic disorders and side effects of spinal anesthesia (SA) on the cardiovascular system. SA-induced sympathetic denervation causes peripheral vasodilatation and redistribution of central blood volume that may lead to decreased venous return to the heart. The aim of the study was to evaluate the changes of inferior vena cava collapsibility index (IVC-CI) during SA in spontaneously breathing patients during elective knee joint replacement surgery to prognose manifestation of intraoperative hypotension and bradycardia. *Materials and methods*: 60 patients (American Society of Anesthesiologists (ASA) physical status I or II, no clinically significant cardiovascular pathology) of both sexes undergoing elective knee joint replacement surgery under SA were included in the prospective study. Inspiratory and expiratory inferior vena cava (IVCin, IVCex) diameters were measured using an ultrasound device in supine position before and immediately after SA, then 15 min, 30 min, and 45 min after SA was performed. The heart rate, along with systolic, diastolic, and mean arterial blood pressures were collected. The parameters were measured at the baseline and at the next four time points. *Results*: There were no significant changes in IVCin, IVCex, and IVC-CI compared to baseline and other time point measurements in hypotensive versus nonhypotensive and bradycardic versus nonbradycardic patients (*p* > 0.05). Changes in IVC diameter do not prognose hypotension and/or bradycardia during SA: the area under the curve (AUC) of the receiver operating characteristic (ROC) curve for IVC-CI at all measuring points was <0.7, *p* > 0.05. *Conclusions*: Reduction in IVC diameters and increase in IVC-CI do not predict hypotension and bradycardia during SA in spontaneously breathing patients undergoing elective knee joint replacement surgery.

## 1. Introduction

Spinal anesthesia (SA) is an effective and safe method of providing intraoperative analgesia during various surgical procedures. This anesthetic technique is important and widely used for operations on the lower abdomen, pelvis, perineum, and lower limbs. Hypotension and bradycardia are the most common hemodynamic disorders and side effects of SA on the cardiovascular system [1]. According to the literature, the incidence of hypotension occurs from 30% to 90% [2,3,4,5]. The incidence of bradycardia ranges from 9% to 74% [6]. Severe hypotension and bradycardia can lead to cardiac arrest. Although the incident of this cardiovascular complication during SA is extremely rare [1,6], it is necessary to find precise and easily accessible methods to predict and avoid these adverse effects.

The mechanism of SA-induced hypotension and bradycardia is still unclear [7]. During SA preganglionic sympathetic nerve fibers are blocked. Sympathetic denervation causes peripheral vasodilatation, a slight decrease in myocardial contractility, and decreased heart rate (HR). Peripheral vasodilatation and redistribution of central blood volume lead to a significant reduction in preload [8]. Hypotension can be caused by decreased cardiac output, reduction of systemic vascular resistance, or decreased HR. It is uncertain which one of these three components has a greater influence on the occurrence of hypotension and bradycardia. We hypothesized that development of hypotension and bradycardia during SA might be related with increased collapsibility of inferior vena cava (IVC). Some studies have shown correlation between central venous pressure (CVP), circulating blood volume, and IVC collapsibility index (IVC-CI) [9,10,11,12].

The aim of the study was to evaluate the changes of IVC-CI during SA in spontaneously breathing patients during elective knee joint replacement surgery to prognose manifestation of intraoperative hypotension and bradycardia (rather than analyzing the relationship between ultrasonography and hemodynamic indices).

## 2. Materials and Methods

The study took place at the Department of Orthopaedics and Traumatology, Hospital of Lithuanian University of Health Sciences Kauno Klinikos, from March 2016 to January 2017. Ethical approval for this study was provided by the Kaunas Regional Biomedical Research Ethics Committee (No. BE-2-5 issued on 18 February 2016).

The inclusion criteria of the patients were as follows: Age ≥ 18 years; written consent to participate in the study; patients undergoing elective knee joint replacement surgery under SA; patients conformed to American Society of Anesthesiologists (ASA) physical status I or II in preoperative assessment; no severe cardiac pathology.

The exclusion criteria were as follows: patients' refusal to participate in the study; other type of anesthesia; ASA III and above; severe cardiovascular pathology; severe intraoperative blood loss requiring blood transfusion; impossible to measure IVC diameter.

The following demographic characteristics of the study were collected: population gender, age, body mass index, body surface area, and ASA status. Routine monitoring of electrocardiography (ECG), noninvasive blood pressure and pulse oximetry were initiated upon arrival to the operating room. Premedication of midazolam 2.5–5 mg was given to all patients and a slow fluid therapy of crystalloids was started once an intravenous cannula was placed. SA was performed in the lateral decubitus position. A 27-G spinal pencil type needle was inserted in the L_3–4_ intervertebral space. After identification of the subarachnoid space, 15–17 mg of levobupivacaine was administered. After completing the subarachnoid injection, patients were positioned in supine position with one leg bended, as this position is needed for the surgery. All doses of medications were adjusted according to the individual patient’s needs. To determine the level of SA, a cold test with alcohol wipe was performed.

### 2.1. Measurements

All measurements of IVC were performed in supine position with one leg bended. The curvilinear probe (Flex Focus 400 exp, bk medical, Denmark 2–6 MHz) was used. The measurements of IVC diameter were taken in M-mode 1–2 cm below the level of the hepatic vein in the long axis subcostal view [13,14,15,16]. IVC-CI was calculated using the following equation: IVC-CI = ((IVCex − IVCin)/IVCex) × 100% [13]. Patients were asked to breathe normally during sonography of IVC, as breathing rate was expressed as breaths per minute (b/min).

HR, systolic (BPs) and diastolic (BPd) arterial blood pressures were recorded. Hypotension was considered as BPs value drops below baseline by more than 30%. Bradycardia was considered as HR lower than 50 bpm. Mean arterial blood pressure (MAP) was calculated using the following equation: MAP = (2 × BPd + BPs)/3. Decreased circulating blood volume was considered as IVC-CI higher than 50% [13]. The parameters were noted at the baseline and at the next four time points. The baseline measurements were performed before SA. Measurements at time point 1 were performed immediately after SA; time point 2, 15 min after beginning of SA; time point 3, 30 min after beginning of SA; and time point 4, 45 min after beginning of SA. All measurements were performed by the same trained investigator.

### 2.2. Statistical Analysis

The statistical analysis was performed using IBM SPSS 22.0 software. The data are presented as mean with standard deviation or median with a range. Kolmogorov–Smirnov test was used to determine normal distribution of the data. Normally distributed variabilities were analyzed using ANOVA test to compare multiple dependent groups (BPs, BPd, MAP, HR, IVC-CI between the time points) and Student t test for analyzing the differences in means between two independent groups (age, body mass index, body surface area, MAP, HR, breathing rate, IVC-CI between the groups). Non-normally distributed variabilities were analyzed using the Friedman test to compare multiple dependent groups (IVCin, IVCex between the time points). The data on the nominal scale were compared using Chi-square test (gender, ASA status). Pearson correlation coefficient was used to demonstrate correlation between the variables. A receiver operating characteristic (ROC) curve was plotted to determine the threshold value of IVC-CI which provided the prediction of hypotension and bradycardia. We assumed IVC-CI to be clinically relevant if the area under the curve (AUC) was >0.7. Statistical significance was determined as *p* < 0.05.

To detect the significant difference in mean values of variability of IVC-CI more than 40% for hypotensive versus nonhypotensive and bradycardic versus nonbradycardic patients assuming significance level alpha = 0.05 and power of the test = 0.8, we should have at least 6 patients in each group. 

## 3. Results

Sixty ASA I–II class patients who were scheduled for elective knee joint replacement surgery and underwent SA were included in the prospective study. There was no significant difference in demographic data between hypotensive versus nonhypotensive and bradycardic versus nonbradycardic patients. Demographic data of the patients is presented in Table 1.

**Figure 1:**
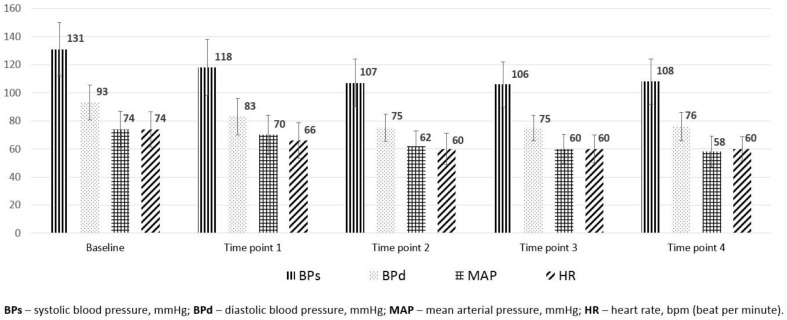
The variations of hemodynamic parameters (systolic and diastolic blood pressure, mean blood pressure, heart rate) during SA. A statistically significant difference was found between all hemodynamic parameters measured at all time points and the baseline measurements (*p* < 0.001).

There was a statistically significant decrease in BPs, BPd, MAP, and HR immediately after the infusion of local anesthetic and all the other time points compared to baseline measurement (*p* < 0.001). The hemodynamic parameters at different time points during SA are shown in Figure 1. Although hypotension during SA was common almost in all patients, severe hypotension (drop of arterial blood pressure >30% from the baseline) was registered in 25% (*n* = 15) of the patients, and bradycardia <50 bpm was seen in 18.3% (*n* = 11) cases.

There were no significant changes in IVCin, IVCex, and IVC-CI compared to baseline and the other time point measurements (*p* > 0.05). The variations of ultrasonography indices are shown in Table 2. No statistically significant difference was detected between changes in IVC-CI at any time point comparing hypotensive versus nonhypotensive and bradycardic versus nonbradycardic patients (*p* > 0.05). The variations of collapsibility of IVC between the groups are shown in Table 3.

There was no correlation between BPs and IVC-CI at any time point: time point 1 (*r* = 0.169, *p* = 0.196); time point 2 (*r* = −0.012, *p* = 0.927); time point 3 (*r* = 0.015, *p* = 0.909); and time point 4 (*r* = −0,089, *p* = 0.499). The same results were comparing relations between BPd and IVC-CI: time point 1 (*r* = 0.246, *p* = 0.058); time point 2 (r = −0.068, *p* = 0.607); time point 3 (*r* = 0.02, *p* = 0.879); and time point 4 (*r* = −0,182, *p* = 0.165). We found no statistically significant correlation between MAP and IVC-CI at any time point: time point 1 (*r* = 0.203, *p* = 0.102); time point 2 (*r* = −0.052, *p* = 0.69); time point 3 (*r* = 0.05, *p* = 0.679); and time point 4 (*r* = −0,133, *p* = 0.309). The same was found comparing the relations of HR and IVC-CI: time point 1 (*r* = −0.052, *p* = 0.694); time point 2 (*r* = −0.233, *p* = 0.073); time point 3 (*r* = −0.117, *p* = 0.372); and time point 4 (*r* = −0.06, *p* = 0.65).

**Figure 2:**
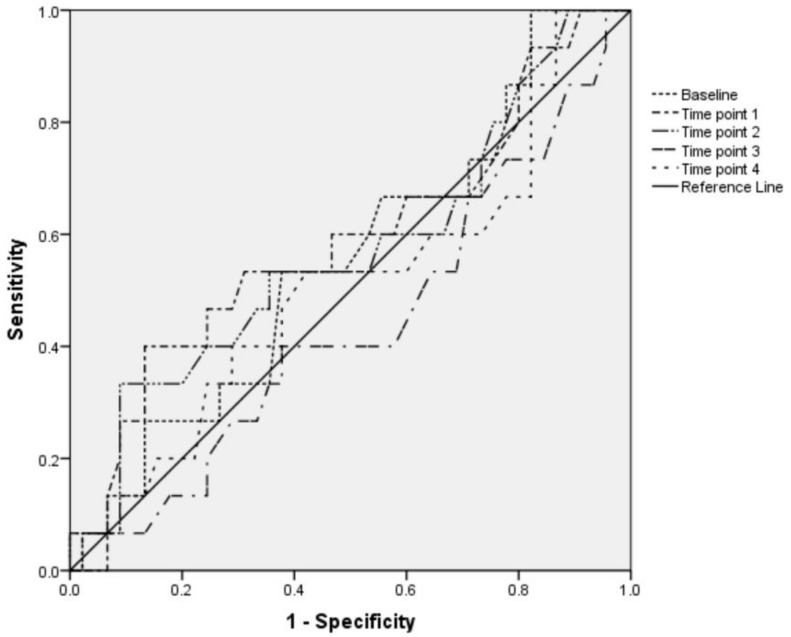
Receiver operating characteristic (ROC) curve analysis of the variations of IVC-CI during SA in elective surgery as predictor of severe hypotension. Area under the ROC curve: Baseline (before SA)—AUC 0.56 (95% CI 0.39, 0.72, *p* = 0.528). Time point 1 (immediately after SA was performed)—AUC 0.59 (95% CI 0.41, 0.76, *p* = 0.314). Time point 2 (15 min after beginning of SA)—AUC 0.57 (95% CI 0.39, 0.75, *p* = 0.417). Time point 3 (30 min after beginning of SA)—AUC 0.44 (95% CI 0.27, 0.61, *p* = 0.489). Time point 4 (45 min after beginning of SA)—AUC 0.51 (95% CI 0.33, 0.69, *p* = 0.918).

**Figure 3:**
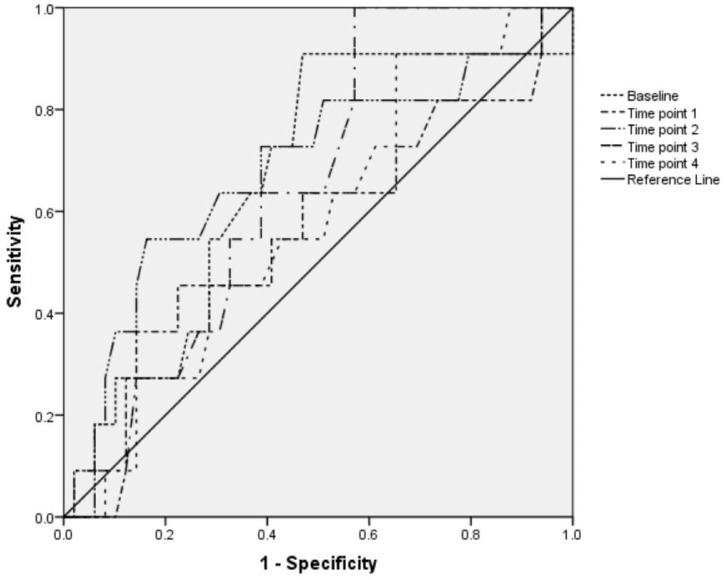
ROC curve analysis of the variations of IVC-CI during SA in elective surgery as predictor of bradycardia. Area under the ROC curve was <0.7. Area under the ROC curve: Baseline (before SA)—AUC 0.67 (95% CI 0.49, 0.84, *p* = 0.082). Time point 1 (immediately after SA was performed)—AUC 0.56 (95% CI 0.36, 0.76, *p* = 0.535). Time point 2 (15 min after beginning of SA)—AUC 0.68 (95% CI 0.5, 0.87, *p* = 0.06). Time point 3 (30 min after beginning of SA)—AUC 0.66 (95% CI 0.51, 0.81, *p* = 0.109). Time point 4 (45 min after beginning of SA)—AUC 0.58 (95% CI 0.41, 0.75, *p* = 0.411).

IVC-CI does not predict hypotension or bradycardia associated with SA: the AUC of the ROC curve for IVC-CI at all measuring points for prognose severe hypotension was less than 0.7 and *p* value was more than 0.05. The AUC of the ROC curve for variations of IVC-CI at all measuring points to predict bradycardia was also below 0.7 (*p* > 0.05). The results are shown in Figure 2 and Figure 3.

## 4. Discussion

Hypotension and bradycardia are not rare during SA [1,4]. It is confirmed in the current study as well. During SA, local anesthetic blocks somatic, motor, sensory, and preganglionic sympathetic fibers. The cardiovascular adverse effects are related to the extent of sympathetic denervation. The sympathetic trunk consists of preganglionic fibers from T1 to L2-3 segments. During SA, cardiovascular response is individual and depends on the high of sympathetic block. The hemodynamic changes are minimal if the block high is below L3 [17]. In our study, the sensory block reached Th10-11 and does not differ between the groups. Sympathetic denervation produced by SA causes peripheral vasodilatation that may lead to hypotension. The hemodynamic changes depend on venous and arterial dilatation induced by sympathetic block [17]. The venodilatation effect dominates. Evaluation of hemodynamic status is important part in the optimization of the fluid therapy. As a result, we can ensure adequate tissue perfusion that depends on cardiac output (CO) and systemic vascular resistance [18]. The changes of blood volume status can be assessed by measuring IVC diameters and calculating IVC-CI [9,10,11,12].

We hypothesized that the development of hypotension and bradycardia during SA might be related to increased collapsibility of IVC which suggest the patients as fluid responder due to relative decreased intravascular volume caused by vasodilatation. Ultrasonography is a noninvasive diagnostic technique that provides continuous information of the hemodynamic status of the patient [13,18]. It is a useful tool for clinical decision-making as extended ultrasound monitoring significantly changes the perioperative patient’s management [19,20,21].

We found the reduction of arterial blood pressure after the injection of local anesthetic to subarachnoid space in all time points compared to the baseline. However, the rate of significant hypotension during SA is lower in our study compared to the literature [2,4] and is 25%, while bradycardia is reported in 18.3% of cases, which was similarly frequent according to the literature [5].

We failed to find statistically significant correlation between ultrasonography indices of IVC and hemodynamic parameters while others show opposite results [22]. Some studies found correlation between CVP, circulating blood volume, and increased IVC-CI [13]. Even though BPs, BPd, MAP, and HR decrease after the beginning of SA, we did not find IVCin, IVCex, or IVC-CI measurements to change significantly at any time point. Furthermore, there was no correlation between changes in arterial blood pressure and HR to changes of IVC-CI. Patil et al. [23] found poor but statistically significant correlation between IVC-CI and BPd and MAP. However, changes in BPs did not correlate to variability of IVC. Our study results suggest the hypotension during SA is not due to relative decrease in intravascular volume. According to the results the occurrence of hypotension during SA may depend on afterload. Drop in arterial blood pressure results primarily from decreased arterial resistance by a blockade of sympathetic nerves.

Zhang et al. [24] state the preoperative ultrasound IVC-CI measurement was a reliable predictor of hypotension after induction of general anesthesia. Au et al. [22] found that patients with IVC-CI ≥50% were more likely to develop significant hypotension associated with administration of propofol. This research suggests IVC variability to be a useful tool to predict patient with a higher risk of hypotension during surgery. Our study shows the opposite results. However, differences in patient population and anesthesia methods among the studies may have resulted in the discrepancy. In our study propofol was not used to eliminate its effect on arterial blood pressure. What is more, opposite results can be a caused of adequate fluid balance before the surgery as our patients were allowed to drink up to 2 h before the surgery. The type of surgery performed is also important as during knee replacement one knee must be bent. As a result, the venous return to the right heart might increase. In our study, all patients in all measurement time point were in the same position.

The present study shows that variations of IVC-CI do not predict hypotension and bradycardia in spontaneously breathing patients during SA in elective knee replacement surgery, as the AUC of the ROC curve for IVC-CI was <0.7. Furthermore, it is important to mention that, in spontaneously breathing patients, respiratory variations of IVC diameter are highly variable from one cycle to another in a particular patient and between different patients [13]. The value of IVC variability or index for intravascular fluid evaluation in spontaneously breathing patients remains the object of discussion [13,14,15,24,25]. Moreover, it is known that breathing manner significantly affects IVC diameter [13,26,27]. Patients were asked to breathe normally and not to perform deep breaths while IVC parameters were measured. Breathing rates were also similar among the patients, i.e., 12–14 times per minute. Pain can cause less change in intrathoracic excursion, which might lead to minor IVC variations. In our case, pain could not have had an effect on breathing manner. As has been mentioned before in the method section, if any pain was present the strategy of the anesthesia was changed and the patient was eliminated from the study.

## 5. Study Limitations

Although the sample size was small (*n* = 60) it was sufficient for statistical calculations. Even though blood loss was not recorded, the patient was eliminated from the study if there was a need for intraoperative blood transfusion due to severe bleeding. Large-scale prospective studies in patients undergoing SA for surgery will be needed to confirm and expand on the finding of the present study, including the use of other methods and clinical parameters in a variety of patient subgroups.

## 6. Conclusions

Reduction in IVC diameters and increase in IVC-CI do not predict hypotension and bradycardia during SA in spontaneously breathing patients undergoing elective knee joint replacement surgery.

It seems SA does not affect circulating blood volume and heart preload by increasing variability of IVC.

## Figures and Tables

**Table 1:** Demographic data of the patients and comparisons between hypotensive vs. nonhypotensive and bradycardic vs. nonbradycardic groups.

	Total	Hypotensive	Nonhypotensive	*p* Value *
Sex, *n* (%) Male Female	14 (23.3) 46 (76.7)	4 (28.6) 11 (23.9)	11 (71.4) 35 (76.1)	0.487
Age, mean (SD), years	69.35 (9.14)	66.13 (7.3)	70.4 (9.5)	0.16
ASA status, *n* (%) I II	8 (100) 52 (100)	2 (25) 13 (25)	6 (75) 39 (75)	0.65
Body mass index, mean (SD), kg/m^2^	30.97 (4.77)	33 (4.1)	30 (4.8)	0.706
Body surface area, mean (SD), m^2^	2.043 (0.182)	2.1 (0.159)	2.01 (0.183)	0.98
MAP before spinal anesthesia (SA), mean (SD), mmHg	93 (12.9)	101 (13.15)	90 (11.72)	0.34
HR before SA, mean (SD), bpm	73 (12.5)	72 (11.4)	74 (12.9)	0.54
Breathing rate, mean (SD), b/min	14 (2)	13 (1.6)	14 (2.5)	0.25
IVC_ex_ baseline, mean (SD), mm	13.9 (5.5)	13.7 (3.9)	13.9 (5.9)	0.911
IVC_in_ baseline, mean (SD), mm	8.9 (4.3)	8.5 (3.3)	9 (4.6)	0.676
IVC-CI baseline, mean (SD), %	35.6 (18.7)	38.4 (18.5)	34.7 (18.8)	0.521
	**Total**	**Bradycardic**	**Nonbradycardic**	***p*** **Value ^†^**
Sex, *n* (%) Male Female	14 (23.3) 46 (76.7)	4 (28.6) 7 (15.2)	10 (71.4) 39 (84.8)	0.258
Age, mean (SD), years	69.35 (9.14)	70.09 (7.4)	69.18 (9.5)	0.769
ASA status, *n* (%) I II	8 (100) 52 (100)	0 (0) 11 (21.2)	8 (100) 41 (78.8)	0.061
Body mass index, mean (SD), kg/m^2^	30.97 (4.77)	33 (4.9)	30 (4.65)	0.715
Body surface area, mean (SD), m^2^	2.043 (0.182)	2.14 (0.171)	2.02 (0.178)	0.727
MAP before SA, mean (SD), mmHg	93 (12.9)	94.39 (11.8)	92.6 (13.3)	0.697
HR before SA, mean (SD), bpm	73 (12.5)	67 (9.1)	76 (11.3)	0.508
Breathing rate, mean (SD), b/min	14 (2)	13 (1.6)	14 (2.5)	0.25
IVC_ex_ baseline, mean (SD), mm	13.9 (5.5)	13 (5.8)	13.9 (5.4)	0.894
IVC_in_ baseline, mean (SD), mm	8.9 (4.3)	7.6 (3.7)	9.2 (4.4)	0.266
IVC-CI baseline, mean (SD), %	35.6 (18.7)	43.7 (18.4)	33.8 (18.5)	0.15

Mean arterial pressure (MAP); heart rate (HR); beats per minute (bpm); b/min, breaths per minute. There were no significant differences between the groups hypotensive vs. nonhypotensive and bradycardic vs. nonbradycardic patients according to demographic data (*p* > 0.05). * *p* value of comparison variabilities between hypotensive vs. nonhypotensive groups. ^†^ *p* value of comparison variabilities between bradycardic vs. nonbradycardic groups.

**Table 2:** The variations of ultrasonography indices.

Parameters	Baseline	Time Point 1	Time Point 2	Time Point 3	Time Point 4	*p* Value
IVCex, median (range), mm	12.5 (5.67–32.2)	13 (6.1–33.1)	13 (6.88–24.8)	13.7 (5.23–22.9)	12.8 (5.23–23.4)	>0.05
IVCin, median (range), mm	8.2 (2.29–22.9)	9.2 (3.03–24.8)	8.7 (2.7–17.4)	8.3 (3.1–19.8)	8.3 (2.5–15.7)	>0.05

Expiratory inferior vena cava (IVCex); inspiratory inferior vena cava (IVCin); inferior vena cava collapsibility index (IVC-CI); baseline, time point before SA; time point 1, immediately after SA was performed; time point 2, 15 min after beginning of SA; time point 3, 30 min after beginning of SA; time point 4, 45 min after beginning of SA. There were no significant changes in IVCin, IVCex, and IVC-CI compared to baseline and other time point measurements (*p* > 0.05).

**Table 3:** The collapsibility of IVC during SA between the groups (hypotensive vs. nonhypotensive, bradycardic vs. nonbradycardic).

Time Point	IVC-CI, %					
	Hypotensive Group	Nonhypotensive Group	*p* Value *	Bradycardic Group	Nonbradycardic Group	*p* Value *
Baseline	38.4 (18.5)	34.8 (18.9)	0.984	43.7 (18.5)	33.8 (18.5)	0.55
Time point 1	35.6 (19.1)	30.22 (17.7)	0.34	35.2 (19)	30.8 (17.9)	0.629
Time point 2	37.9 (21.2)	31.16 (16.2)	0.104	41.7 (18.5)	30.8 (17)	0.578
Time point 3	29.4 (18.4)	32.13 (16.7)	0.794	38.2 (13.7)	29.9 (17.5)	0.25
Time point 4	32.6 (17.2)	30.93 (17.4)	0.878	34 (17.9)	30.8 (14.5)	0.632
*p* value ^†^	>0.05	>0.05		>0.05	>0.05	

Values are mean (standard deviation). IVC-CI; Baseline, time point before SA; time point 1, immediately after SA was performed; time point 2, 15 min after beginning of SA; time point 3, 30 min after beginning of SA; time point 4, 45 min after beginning of SA. There were no significant changes in IVC-CI compared to baseline and other time point measurements in groups and between groups (hypotensive vs. nonhypotensive, bradycardic vs. nonbradycardic) at all other time points (*p* > 0.05). * *p* value of comparison IVC-CI between groups (hypotensive vs. nonhypotensive, bradycardic vs. nonbradycardic) at all time points. ^†^ *p* value of comparison IVC-CI between baseline and other time point in groups.
